# Correlation among post-surgery recurrence of CRSwNP and TCM syndromes and tissue inflammatory cell infiltration type: a study protocol

**DOI:** 10.1186/s13643-024-02562-9

**Published:** 2024-05-30

**Authors:** Yan Xie, Fangqi Liang, Li Zhou, Qing Chen, Feifei Chen, Qinwei Fu, Shiqi Wu, Dandi Zeng, Wanling Cui, Linzhi Liao, Luyun Jiang

**Affiliations:** 1 Department of Otorhinolaryngology, Hospital of Chengdu University of Traditional Chinese Medicine, No. 39, Shierqiao Road, Jinniu District, Chengdu, Sichuan Province 610075 People’s Republic of China; 2 Department of Otolaryngology, Chengdu Integrated TCM and Western Medicine Hospital: Chengdu First People’s Hospital, Chengdu, Sichuan Province 610095 People’s Republic of China; 3 Chengdu University of Traditional Chinese Medicine, No. 37, Shierqiao Road, Jinniu Distric, Chengdu, Sichuan Province 610075 People’s Republic of China

**Keywords:** CRSwNP, Traditional Chinese medicine, Issue inflammatory cell infiltration type, Prognosis, Study design

## Abstract

**Background:**

Functional endoscopic sinus surgery is a principal option for treating chronic rhinosinusitis with nasal polyps (CRSwNP) after medication failures. Unfortunately, some patients still have unsatisfactory postoperative recovery. The type of inflammatory cell infiltration in nasal polyp tissue has been reported available for recurrence prediction. As it is invasive and time-consuming, this technique is hard to promote clinically under the existing technical conditions. And during the course of clinical treatment, we have noted that differences in the postoperative recurrence rate of patients present among different traditional Chinese medicine syndrome types.

**Methods and analysis:**

This is a non-randomized, single-center, and prospective cohort study started in Chengdu Sichuan Province, People’s Republic of China, in January 2021. A total of 200 participants will be recruited from patients who are diagnosed with CRSwNP and prepared for functional endoscopic sinus surgery. We collect preoperative data which includes general information, medical history, TCM syndromes, visual analogue scale (VAS) of subjective symptoms, Lund-Kennedy endoscopic score, and Lund-Mackay score of computed tomography (CT) scanning of sinuses. We acquire the VAS score and Lund-Kennedy score of subjective symptoms through multiple planned follow-up after surgery. After 1 year of follow-up, the recurrence rate will be calculated, and the curative effect will be assessed. Meanwhile, the patients’ pathological sections will be sorted out, and inflammatory cell infiltration will be analyzed. Statistical analysis will be carried out to evaluate the correlation among CRSwNP recurrence and TCM syndrome types and tissue inflammatory cell infiltration types. Then we will establish a predictive model for CRSwNP recurrence. Analyses of survey data include descriptive and inferential statistical approaches.

**Discussion:**

This is the first prospective cohort study on investigating the correlation of CRSwNP recurrence with TCM syndrome types and tissue inflammatory cell infiltration types. Through this study, we hope to discover a new and simple, effective, and noninvasive way to predict the recurrence rate rapidly after CRSwNP and provide reference for the intervention timing of traditional Chinese medicine application, thereby achieving customized diagnosis and treatment, minimizing risks of surgical events, and delaying postoperative recurrence of CRSwNP.

**Systematic review registration:**

PROSPERO ChiCTR2100041646.

## Background

Chronic rhinosinusitis with nasal polyps (CRSwNP) refers to the chronic inflammation of the sinuses and nasal mucosa, accompanied by nasal polyps. The course of the disease exceeds 12 weeks. The clinical symptoms include nasal congestion, purulent nasal discharge, sense of smell disorders, headache, and intranasal polyps [1, 2]. Functional endoscopic sinus surgery (FESS) is the major method for clinical treatment of CRSwNP when maximal drug therapy fails [3]. With the extensive development of FESS and the gradual maturity of technology and equipment, the clinical cure rate of CRSwNP has gradually improved. Despite this, there are still about 30 to 60% of patients who complain about the unsatisfactory post-surgery recovery, residual nasal discharge, and no recovery of smell. Relevant studies by DeConde A. S. have found that the recurrence rate of CRSwNP is as high as 40% at 18 months after surgery [4]. A 2-year follow-up of CRSwNP in the Chinese population has indicated the recurrence rate of 55.6% [5].

Several recent studies have shown an intimate association between the type of inflammatory cell infiltration in nasal mucosal polyps and postoperative recurrence [6–8]. The intrinsic pathological features of eosinophilic and non-eosinophilic nasal polyps affect greatly on surgical effect and post-surgery recurrence [7, 9]. Wang et al. have reported that the accuracy of eosinophil (EOS) in predicting post-surgery recurrence can be as high as 90% [7]. This is helpful for both surgeons and patients to have a more accurate judgment for the results of surgery, and corresponding individualized management plans can be designed as per the prognosis, laying a foundation for the realization of CRSwNP precise treatment. However, sufficient polyp tissues are hard to be collected when the patient is conscious, plus cell counting is time-consuming; it is therefore difficult to promote extensive clinical application under current conditions.

There is a concept of “syndrome” in traditional Chinese medicine (TCM), known as “ZHENG,” which is an important part of the theory of TCM [10, 11]. Syndrome differentiation is one of the most important concepts in the practice of TCM and comprises a series of diagnostic procedures. Syndrome differentiation is the process of comprehensive analysis of clinical information obtained by the four main diagnostic TCM procedures: observation, listening, questioning, and pulse analyses [12].

The information obtained from traditional Chinese medicine syndrome differentiation includes symptoms, pulse patterns, and tongue patterns, which are considered subjective. However, compared to other prediction tools, traditional Chinese medicine syndrome has advantages because it does not require complex and time-consuming laboratory test for clinical prognosis or survival prediction. Scholars have found that the differentiation and classification of traditional Chinese medicine syndromes have certain significance in predicting the survival of advanced cancer patients [13]. The prognosis of primary liver cancer patients with syndrome of liver depression and spleen deficiency is the best, while the prognosis of syndrome of yin deficiency in the liver and kidney is bad [14].

Based on the main symptoms, accompanied symptoms, tongue and pulse conditions, and local examination characteristics of patients in traditional Chinese medicine, the *Otorhinolaryngology of TCM* classifies the disease into four traditional Chinese medicine (TCM) syndrome types: damp heat in the spleen and stomach, stagnant heat in gallbladder, deficiency and weak in spleen qi, and deficiency and cold in lung qi under the principles of *Otorhinolaryngology in Traditional Chinese Medicine*. In the course of clinical treatment, we have noted that the recurrence rate of patients with different TCM syndromes varies to some extent [15–18]. Previous research have indicated that the 1-year postoperative recurrence rate in patients with the types of deficiency and weak in spleen qi, and deficiency and cold in lung qi, is higher than that of patients with damp heat in the spleen and stomach and stagnant heat in gallbladder [17, 18]. After FESS surgery, although there are many factors that affect the prognosis, such as the exposure stage after discharge, living and dietary environment, and the patient's selfdiscipline. It is certain that the postoperative recurrence rate of the first two TCM syndromes is markedly higher than that of the latter two types, which occurs not an isolated case but according to some regular rules.

Since both TCM syndromes and tissue inflammatory cell infiltration are associated with the recurrence rate of CRSwNP, do the characteristics of tissue inflammatory cell infiltration correlate with TCM syndrome types? That is, there are differences in the inflammatory cell infiltration of CRSwNP in different TCM syndrome types, and this difference is a key factor influencing the postoperative recovery of CRSwNP. Hence, we designed a non-randomized, single-center prospective cohort study to explore the correlation among the recurrence rate of CRSwNP and TCM syndrome types and the characteristics of tissue inflammatory cell infiltration. Through this study, we hope to discover a new and simple, effective, and noninvasive way to predict the recurrence rate rapidly after CRSwNP and provide a direction for thinking about the timing of intervention in traditional Chinese medicine, thereby achieving customized diagnosis and treatment, minimizing risks of surgical events, and delaying postoperative recurrence of CRSwNP.

## Methods/design

### Design overview

This study is a non-randomized, single-center, prospective cohort study, starting in January 2021, and it is currently ongoing. A total of 200 patients at 12–75 years of age who are prepared to undergo FESS will be recruited from Hospital of Chengdu University of Traditional Chinese Medicine.

Due to the similar degree of improvement in endoscopic scores and quality-of-life indicators among patients undergoing chronic sinusitis surgery at different age groups, or without exceeding the minimum clinically important differences [19–21], this study did not consider further age stratification factors. All participants are diagnosed with CRSwNP. And preoperative data will be collected, as well as follow-up visits and relevant data collection after surgery. All participants should be advised to undergo regular follow-up examinations and treatments at the hospital after surgery, regardless of their participation in our study. We are simply following up on patients.

The participants will be scheduled with follow-up at 2 weeks, 4 weeks, and 2, 3, 6, 9, and 12 months after surgery. The included patients will be assigned to different cohorts in light of corresponding TCM syndrome types and completed the 1-year-long cohort research. Before the start of this study, all researchers were trained to get familiar with the criteria of awarding scores, to improve the observation consistency of the researchers, and to ensure the reliability of the clinical research conclusions. The feasibility and accuracy of the entire process were overseen by two independent supervisors, who were responsible for inspection and confirmation of all records and reports of study data and case reports filled in correctly and completely, as well as the consistence with the original data. To ensure the follow-up visits of patients as scheduled, we will remind patients of their planned visit time via follow-up schedules, phone calls, or text messages.

### Study settings and participants

This study is still ongoing at Affiliated Hospital of Chengdu University of Traditional Chinese Medicine, and the identification of TCM syndrome types of the participants will be carried out by professional experts with TCM background. Regarding the data quality assurance of traditional Chinese medicine syndrome identification, the identification of traditional Chinese medicine syndrome types by participants will be carried out by two experts. In case of inconsistency, a third expert will be invited to join, and the final conclusion will be drawn after discussion among the three experts. The present study categorized the participants four TCM syndrome types: stagnant heat in gallbladder, damp heat in the spleen and stomach, deficiency and cold in lung qi, and deficiency and weak in spleen qi under the guide of *Otorhinolaryngology in Traditional Chinese Medicine* (Table 1) as Cohort 1, Cohort 2, Cohort 3, and Cohort 4. For eligible patients, investigators have detailed interpretation on purpose, specific content, and the designed methods on of how to complete the trial. Patients will be included in the study after the signature of informed consent.

**Table 1:** Classification of TCM syndrome criteria

TCM syndrome types	Main symptoms	Accompanied symptoms	Tongue and pulse condition	Local examination
Stagnant heat in gallbladder	Nasal obstruction, decreasing sense of smell, bad headache, getting nasal purulence, in yellow or yellow green, in a large amount, or in odor	Whiny and irritability, mouth bitterness, throat drying, vertigo, tinnitus deafness, poor sleep	The tongue is red with yellow or greasy fur, and the pulse is rapid and profound or uneven and profound	Polyps are grayish or pale or dark red or easily bleeding and soft or tough
Damp heat in the spleen and stomach	Nasal obstruction, decreasing sense of smell, headache, runny nose, and yellow nasal discharge in a large amount	Distension and fullness in epigastric and abdominal areas, eating less, weakness and fatigue, viscous excrement	The tongue is red with yellow and greasy fur, and the pulse is rolling and rapid	Polyps are grayish or pale or dark red or easily bleeding and soft or tough
Deficiency and cold in lung qi	Nasal congestion or light or heavy, decreasing sense of smell, lightheadedness, runny nose which is white and sticky	Onset of sneezing, shortness of breath and fatigue, low faint voice, spontaneous sweating and chills from self	The tongue is pale with thin and white fur, and the pulse is weak and slow	Polyps are white or pale in color and soft
Deficiency and weak in spleen qi	Nasal congestion, decreasing sense of smell, lightheadedness, runny nose, runny or yellow white facies	Weakness and fatigue, eating less, abdominal distension, and loose stools	The tongue is fat with dentate marks, the fur is thin and white or white and greasy or little, and the pulse is wiry and weak	Polyps are white or pale in color and soft

**Fig. 1:**
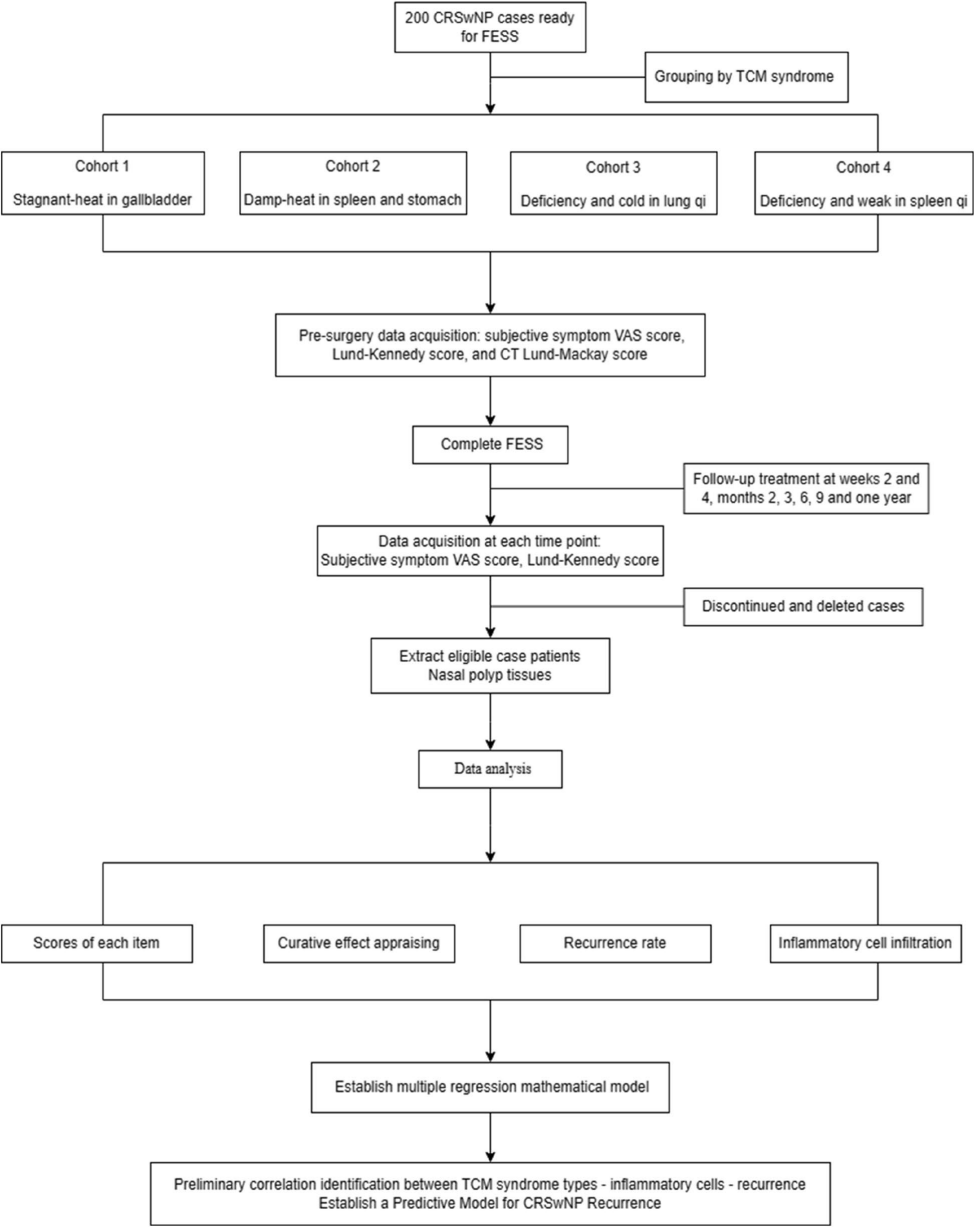
Flow chart of the study

**Fig. 2:**
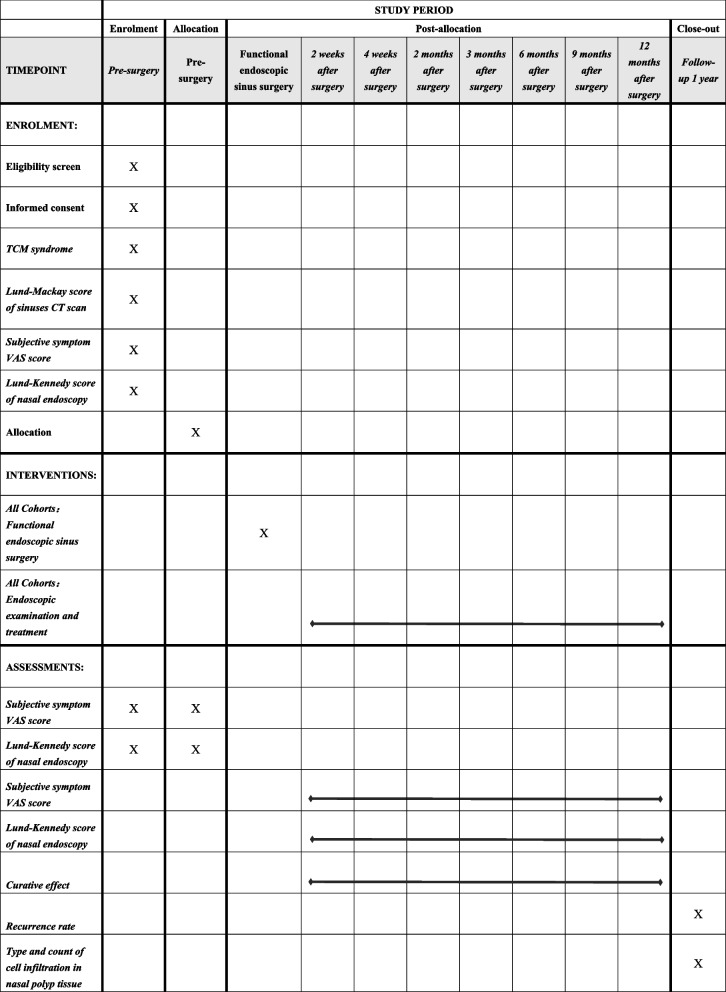
SPIRIT schedule of enrollment, interventions, and assessments

After being included in the study, preoperative data of all participants will be collected, including general information, medical history, TCM syndromes, VAS score of subjective symptoms, Lund-Kennedy score of nasal endoscopy, and CT scan Lund-Mackay score of sinuses. Furthermore, follow-up visits were scheduled at weeks 2 and 4 and months 2, 3, 6, 9, and 12 after surgery, and the subjective symptom VAS score and the Lund-Kennedy score will be also obtained. After the 1-year follow-up, pathological sections of the patients who completed the trial will be obtained, and the infiltration of inflammatory cells will be analyzed under a 400-fold light microscope. Then, the recurrence rate and EOS count percentage of each TCM syndrome type will be applied, and a multiple regression model will be initially established to explore the correlation among postoperative recurrence of CRSwNP and TCM syndrome types and tissue inflammatory cell infiltration types. Finally, we will establish a predictive model for CRSwNP recurrence. The flow chart of the study is presented in Fig. 1. The SPIRIT schedule of enrollment, interventions, and assessments is presented in Fig. 2.

If any participant discontinues follow-up visits, a serious adverse event (AE) occurs, any traditional Chinese medicine treatment is applied which might affect their prognosis during the follow-up period, or the investigator determined that it is inappropriate for the participant to continue the trial for safety reasons, this participant is withdrawn from the current research.

#### Inclusion criteria

Subjects are eligible to participate if they meet the following criteria: the patients are as follows:

1. Meet the diagnostic criteria of European CRSwNP 2020 [1] and Chinese CRSwNP 2018 [2].
2. Age between 12 and 75 years old (including 12 and 75 years old) and no gender limit
3. Are ready to undergo FESS
4. Are able to perform regular routine postoperative treatment
5. Are able to independently complete the questionnaire of this research
6. Are willing to participate in this trial and sign the informed consent form

#### Exclusion criteria

Subjects will be excluded if they meet any of the following criteria:

1. Patients who have allergic fungal sinusitis, posterior nostril polyps, cysts, parasitic infections, autoimmune diseases, immunodeficiency diseases, severe systemic diseases, and mental illness
2. Patients who have a history of desensitization therapy
3. Patients who have accepted antibiotics, glucocorticoids, and antihistamines treatments within 4 weeks before surgery
4. Pregnancy or lactation

### Outcome measurements

Our researchers are responsible for follow-up of patients before surgery and at weeks 2 and 4 and months 2, 3, 6, 9, and 12 after surgery. After completing the 1-year follow-up, the recurrence rate will be calculated. Meanwhile, pathological sections of the patients who completed the trial will be analyzed, and the infiltration of inflammatory cells will be visualized under a 400-fold light microscope. All registered patients need to adhere to a 1-year follow-up plan before their data can be used for analysis.

#### Primary outcome

The primary outcome of this study is to statistically analyze the recurrence rate 1 year after FESS and its correlation with traditional Chinese medicine syndrome types and eosinophil infiltration, in order to establish a predictive model for CRSwNP recurrence.

The definition of recurrence is that mucosal lesions (nasal polyps, mucus purulent discharge, and/or mucosal inflammation and edema) are seen under nasal endoscopy, the symptoms persist for 1 month without any alleviation, and maximum drug treatment is required [22].

#### Secondary outcomes

Scores and efficacy assessments will be used as secondary outcome indicators in this trial, to judge whether timely drug treatment and necessary secondary surgical intervention will be needed.

The efficacy assessment criteria referred to the *European Position Paper on Rhinosinusitis and Nasal Polyps (EOPS)* 2020 [1] and the Chinese guidelines for diagnosis and treatment of chronic rhinosinusitis (2018) [2]: nasal congestion, runny nose, head and face pain, hyposmia, sleep disturbance, abnormal findings in nasal endoscopy, and drug maintenance treatment are still needed. The presence of any one of the above symptoms is considered as partial control of the disease, three or above conditions as conditions uncontrolled, and free from any one symptom as complete control of the disease.

### Safety assessments

The present study prospectively explored the correlation among postoperative recurrence of CRSwNP and TCM syndrome types and tissue inflammatory cell infiltration types, mainly collecting the data of patients for analysis, and cells of pathological sections will be calculated. There will no specially designed intervention in this study, and we will simply follow the real-world intervention.

### Sample size calculation

This study includes a sample size of 200 cases. The sample size was calculated according to the recurrence rate of different TCM syndrome groups.

There are 4 exposure factors of interest in this study. In the previous study [17], the expected relative risk of the 3 exposure factors was obtained from 71 patients, and then the sample size was calculated according to each of the 3 exposure factor groups, and then the maximum of these was taken as the final sample size.

Preliminary research on four types of traditional Chinese medicine syndromes shows that the recurrence rate (relative risk) of the syndrome of spleen qi deficiency group is Pmax = 11/26 = 42.3%, the recurrence rate (relative risk) of the syndrome of dampness and heat in the spleen and stomach group is 2/32 = 6.25%, and the recurrence rate (relative risk) of the syndrome of stagnated heat in the gallbladder group is Pmin = 0/13 = 0% [17]. According to the *α* = 0.05, *β* = 0.2, and with a sample ratio of 1:2 exposed to a certain TCM syndrome characteristic group and not exposed to a certain TCM syndrome characteristic group, the sample size of each exposure factor group was calculated using PASS 11 software, following the method of sample size calculations in clinical research. Considering that the dropout rate of the research subjects is 10%, we will select the group with the largest appeal sample size. That is, the minimum sample size required for each group is 20 in the exposed group and 40 in the nonexposed group. So, to complete the observation study of four exposure factor groups, the minimum total sample size is 80 cases. The total sample size of this study is 200 cases, which meets the research requirements. It should be noted that at least 20 patients exposed to each type of traditional Chinese medicine syndrome feature need to be included in the sample. At the same time, 200 cases met the sample size of unconditional logistic regression.

### Statistical analysis

SPSS 23.0 software is used for data analysis.

1. For quantitative data, *t*-test and nonparametric test are used to evaluate the pairwise difference between normally distributed data and non-normally distributed data, respectively. For qualitative data, chi-square test is applied for analysis. *P* < 0.05 is considered statistically significant.
2. Apply logistic regression model to evaluate the relative risk of each variable and its correlation with nasal polyp recurrence.
3. Different traditional Chinese medicine syndromes are entered into a regression model, and the predictive value is determined by the area under the ROC curve (AUC). AUC > 0.9: high predictive value of the model; 0.7 < AUC ≤ 0.9: moderate predictive value. When 0.5 < AUC ≤ 0.7, the optimal diagnostic cut-off point is obtained when the Youden index (sensitivity + specificity − 1) of predictive value is maximized.

## Discussion

CRSwNP is the chronic inflammatory disease of sinuses and nasal mucosa, accompanied by nasal polyps. FESS has the advantages of surgical precision, small trauma, and removal of diseased tissues while preserving normal tissues and structures, making it the main surgical modality for the clinical treatment of chronic rhinosinusitis-nasal polyps when drug therapy is ineffective. Notably, new lesions developed in the postoperative luminal mucosa transition stage, including secondary infection, mucosal edema, vesicles, and polyp regeneration, are possible important factors of postoperative recurrence.

Whether it is drug therapy or surgical treatment, a scientific method for evaluating efficacy should be based on objective examination results that are compared with the symptoms and signs before treatment. The evaluation of CRS efficacy is divided into two aspects: subjective evaluation and objective evaluation. EPOS2020 [1] and the Chinese Guidelines for the Diagnosis and Treatment of Chronic Nasal Sinusitis (2018) [2] suggest subjective evaluation using the visual analogue scale (VAS) scoring method and objective evaluation using the Lund-Kennedy scoring method for nasal endoscopy and Lund-Mackay scoring method for sinus CT examination. Studies have shown that VAS scores can not only be used to assess the severity of CRS but also guide perioperative management of CRS, thereby better improving the cure rate of CRS [23]. In 1995, Lund and Kennedy proposed the Lund-Kennedy (LK) endoscopic scoring system, which is based on polyps, edema, secretions, scars, and scabs, and have a certain correlation with symptom scores [24, 25]. The Lund-Mackay score is a method of quantifying the severity of chronic sinusitis through sinus CT examination. Through computed tomography, the pathological condition of sinusitis can be more accurately determined [26].

Wen W. et al. have noted that the type of inflammatory-infiltrating cells in polyp tissue directly affects the strategy of postoperative nasal mucosa maintenance medication, which will directly affect the effect of postoperative recovery [27]. Wang Chengshuo et al. have reported that EOS infiltration in nasal mucosa and polyp tissue is closely related to postoperative recurrence. When the EOS ratio is greater than 27%, the probability of postoperative recurrence can be as high as 80%, and the accuracy of EOS in predicting postoperative recurrence can reach as high as 90% [7] . Unfortunately, as predicting recurrence by EOS count is invasive and time-consuming, it is a challenge facing in clinical implementation.

Some studies have indicated that certain differences in postoperative recurrence rates exist among different TCM syndromes of patients [15–18]. Our previous study has found that TCM syndrome type is one of the influencing factors for the postoperative curative effect of CRSwNP, and the type of deficiency and weak in spleen qi had the worst curative benefit. Furthermore, the degree of EOS infiltration differed in the tissues of CRSwNP patients with different TCM syndrome types, and this type presented the highest degree of EOS infiltration [17, 18].

The information obtained from traditional Chinese medicine syndrome differentiation includes symptoms, pulse patterns, and tongue patterns, which are considered subjective. But the use of traditional Chinese medicine syndrome manifestations for clinical prognosis or survival prediction has significant advantages, as it does not require complex and time-consuming laboratory test values.

Corticosteroids are commonly used drugs in postoperative clinical practice for CRSwNP patients. Long-term or short-term hormone therapy can minimize EOS and postoperative recurrence. Chen Jinming et al. have discovered that the cavity accepted CRSwNP surgery with long-acting hormone therapy can effectively reduce the recurrence rate of CRSwNP and improve the cure rate [28]. Wang Chengshuo et al. have also reported that short-term treatment of nasal polyps with budesonide inhalation suspension by nasal atomization inhalation can rapidly improve nasal symptoms and reduce the volume of polyps, implying it can be used as a preoperative endoscopic treatment for EOS CRSwNP patients [29].

However, as a commonly introduced drug in clinic, glucocorticoid is one of the important pathogenic factors of nontraumatic femoral head necrosis [30]. Nasal glucocorticoids may cause side effects, namely epistaxis and perforation of the nasal septum, and the incidence of epistaxis has been reported to be as high as 20% in prolonged use of intranasal hormones over 1 year [31, 32]. However, hormones are an effective but helpless option to reduce the recurrence of CRSwNP and to control symptoms during recurrence.

Numerous studies have shown that the intervention of traditional Chinese medicine in the perioperative period can reduce the postoperative reaction of CRSwNP, minimize the reaction in the postoperative recovery stage, and help recurrence control [33–36]. We assume that the intervention of traditional Chinese medicine after surgery is necessary.

However, in clinical practice, it is very difficult to introduce the intervention of traditional Chinese medicine for every patient after surgery. The reason is mainly due to the economic burden that both patients and surgeons are not able to perform well. After FESS surgery, patients are required to perform postoperative endoscopic examination for about 1 year. Patients whose symptoms are not controlled or partially controlled need a longer period of drug treatment. If the intervention of traditional Chinese medicine is introduced at the same time, the patient is under an increased economic stress, thus leading to lower patient compliance. Meanwhile, as surgeons do not have an accurate prediction on which TCM syndrome type is likely or inevitably to relapse after surgery, they will also take into account the uncertainty in the ratio of the patient’s economic cost to benefit when prescribing traditional Chinese medicine. Consequently, the suggestions for Chinese medicine intervention treatment are discontinued.

The current study is expected to identify that patients with a certain type of TCM syndrome are more likely or inevitably to relapse after FESS, and it is related to EOS, which has currently been explored more frequently than others, hoping to discover a new, effective, noninvasive, and fast way to predict the recurrence rate after CRSwNP as simple as possible. Taking this as a starting point, targeted traditional Chinese medicine intervention is carried out in the perioperative period as a synergistic means of hormone therapy; Moreover, while reducing unnecessary waste of medical resources, it strengthens the confidence of patients and medical staff in introducing the application of traditional Chinese medicine intervention.

Taken together, we prospectively investigated the correlation between recurrence and TCM syndrome types and tissue inflammatory cell infiltration types by exploring the TCM and Western medicine factors of CRSwNP recurrence. The results of the current trial might be helpful for the discovery of a new, effective, noninvasive, simple, and rapid way to predict the recurrence rate after CRSwNP and provide support for integrated traditional Chinese and Western medicine treatment against CRSwNP. The possibility of this study suggested that as a synergistic means of hormone therapy, targeted TCM intervention in the perioperative period might reduce the recurrence rate after CRSwNP surgery, improve the symptoms of patients and their quality of life, and minimize subsequent burdens on the society and economy.

Although the study is observational, it basically adheres to the Standard Protocol Items: Recommendations for Interventional Trials (SPIRIT) statement.

## Data Availability

No data are available.

## Abbreviations

AE: Adverse event
AUC: Area under curve
CRSwNP: Chronic rhinosinusitis with nasal polyps
CT: Computed tomography
EOS: Eosinophil
EOPS: The European Position Paper on Rhinosinusitis and Nasal Polyps
FESS: Functional endoscopic sinus surgery
ROC: Receiver operating characteristic curve
TCM: Traditional Chinese Medicine
VAS: Visual analogue scale
